# Revisional Surgery After Bilio-Pancreatic Diversion: Indications, Outcomes, and Complications in a High-Volume Center

**DOI:** 10.1007/s11695-026-08680-8

**Published:** 2026-04-24

**Authors:** PAOLO BOCCANELLI, ANTONIO VITIELLO, Domenico Benavoli, Michela Campanelli, Giovanna Berardi, Vincenzo Pilone, Paolo Gentileschi

**Affiliations:** 1 Maria Cecilia Hospital, Cotignola, Italy; 2 University of Rome Tor Vergata, Rome, Italy; 3 University of Naples Federico II, Naples, Italy

**Keywords:** Bilio-pancreatic diversion, Revisional surgery, Bariatric surgery, Weight regain, Malnutrition

## Abstract

**Background:**

Bilio-pancreatic diversion (BPD), once a prominent bariatric procedure, has seen a marked decline in use due to high rates of nutritional deficiencies and frequent need for surgical revisions. Despite this, BPD remains in practice in select centers, leading to a persistent cohort of patients requiring revisional surgery.

**Methods:**

We conducted a retrospective review at a high-volume bariatric surgery center to identify all patients who underwent revisional surgery following primary BPD between January 2015 and December 2025. Inclusion criteria were age ≥ 18 years, documented primary BPD, and complete clinical data. Demographics, surgical details, indications for revision, types of revision, and outcomes were analyzed.

**Results:**

Forty-six patients underwent revision after Scopinaro BPD. Malnutrition/malabsorption (47.8%) and weight regain (37.0%) were the leading indications. Common tract lengthening and gastric pouch resizing were the most frequent revision types. Post-revision, mean weight and BMI decreased further, and gastrointestinal symptoms improved significantly: diarrhea (80.0% to 4.4%), flatulence (90.9% to 20.5%), and abdominal pain (45.5% to 0%). Post-revision surgical complications occurred in 19.6% of patients, most commonly intestinal occlusion (6.5%), with other serious events including volvulus, anastomotic dehiscence, splenic necrosis with pancreatic fistula, perianastomotic hematoma, jejuno-jejunal leak requiring open abdomen, and evisceration with dehiscence (each 2.2%). Most complications required laparotomy, and one patient (2.2%) died due to a jejuno-jejunal leak.

**Conclusions:**

Both malnutrition and weight regain are leading causes for revision after BPD. Revisional surgery is technically demanding but can be performed laparoscopically. However, it should be reserved for experienced centers, as complications following revision are often serious, frequently requiring laparotomy and, in some cases, may require a second intervention or result in mortality. These findings underscore the need for careful patient selection and long-term follow-up after BPD.

## Introduction

The BPD was first introduced in the late 1970 s as a modification of the Roux-en-Y gastric bypass, initially tested in animals and then in humans [1]. However, over the past fifty years, BPD has become one of the least performed bariatric and metabolic procedures worldwide [2] due to high rates of nutritional deficiencies and frequent need for surgical revisions or reversals [3].

In the 1990 s, the procedure was adjusted in two ways. First, the distal gastrectomy was replaced with a sleeve gastrectomy combined with a duodenoileostomy, which preserved the function of the pylorus. Second, the length of the common channel was increased from 50 to 100 cm to enhance the effects of bile and pancreatic juices. This modified approach became known as the biliopancreatic diversion with duodenal switch [4].

For all these reasons, leading experts have even suggested that the BPD could be permanently discontinued [5].

Nevertheless, some groups—particularly in Italy—have continued to perform BPD, initially using open surgery and later adopting laparoscopic techniques. As a result, many patients still live with both the advantages and the challenges associated with this intervention [6].

Aim of this study was to retrospectively evaluate the number of Scopinaro biliopancreatic diversion procedures that required revision at our center in the last decade, with a specific focus on the underlying indications for revision and the types of revisions performed.

## Materials and Methods

A retrospective search was carried out at a high-volume bariatric surgery center (caseload > 1000) with the aim of determining the number and characteristics of revisional procedures performed after primary Scopinaro biliopancreatic diversion (BPD). All patients who underwent revisional surgery following BPD between January 2015 and December 2025 were identified through institutional surgical databases and electronic medical records. Inclusion criteria were: age ≥ 18 years at the time of revision, documented history of primary BPD performed at the center or referred from other institutions, and availability of complete clinical and surgical data regarding both the initial and revisional procedures.

For each patient, the following data were collected: demographic information, surgical approach of the primary BPD (laparoscopic or open), indications type and technique for revision, and any subsequent re-revisions. Mortality events, postoperative complications and gastrointestinal symptoms evolution were also recorded. Data extraction and verification were performed independently by two investigators.

Descriptive statistics were used to summarize patient characteristics, indications for revision, types of revisional procedures, and outcomes. Continuous variables were reported as mean, median, and range; categorical variables as counts and percentages. Paired categorical outcomes were compared using McNemar’s test, while continuous non-normally distributed variables were assessed using the Wilcoxon signed-rank test for paired samples. All analyses were performed using standard spreadsheet and Microsoft 356 premium.

## Results

A total of 46 patients underwent revisional surgery following biliopancreatic diversion (BPD) at our institution.

The mean age at revision was 50.2 years (median 48.0, range 37.0–70.0). The mean interval between the primary intervention and revision was 17.9 years (median 19.0, range 2.0–30.0). The year of the primary intervention ranged from 1992 to 2016, while the year of revision ranged from 2015 to 2025.

### Weight and BMI Changes

**Fig. 1:**
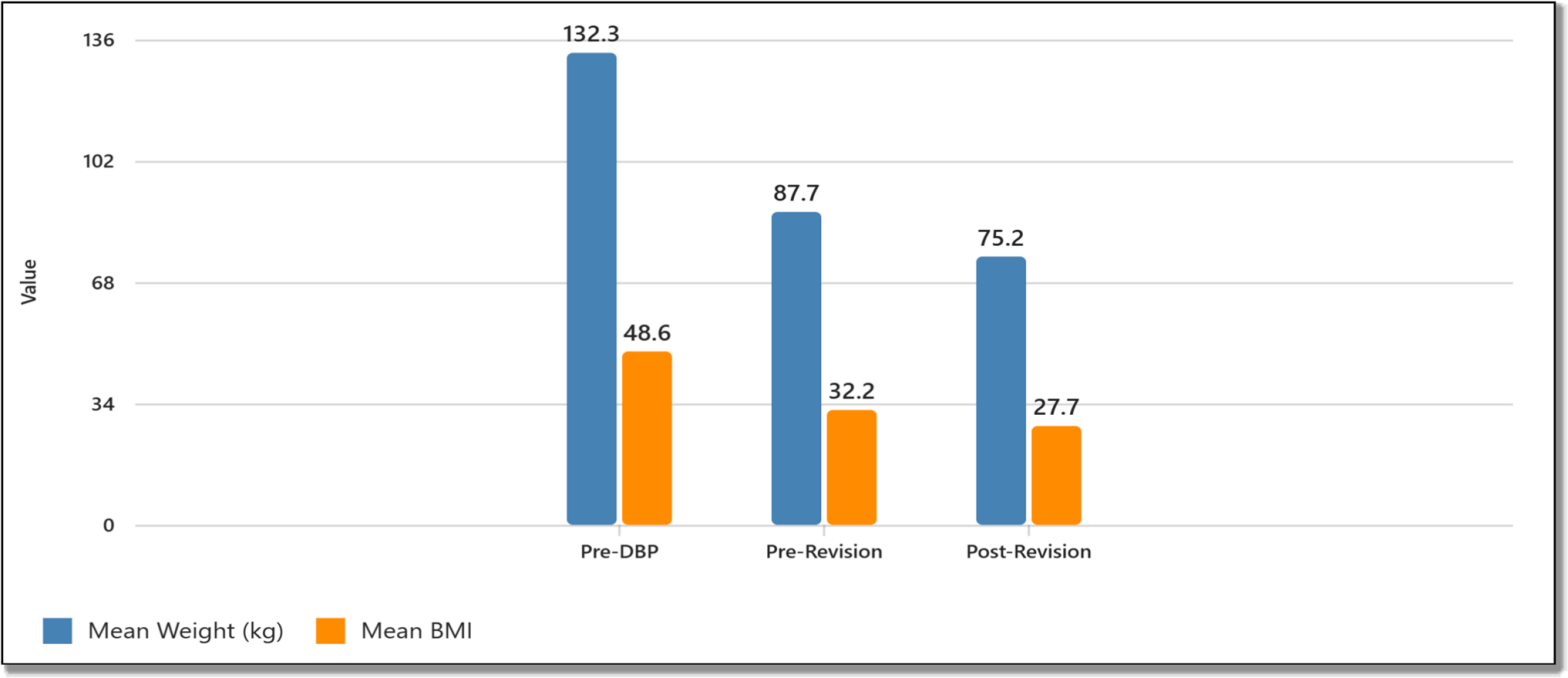
Comparison of mean weight (kg) and BMI across three stages: pre-BPD, pre-revision, and post-revision

The mean pre-BPD weight was 132.3 kg (median 130.0, range 93–242), with a corresponding BMI of 48.6 kg/m2 (median 46.5, range 37.6–86.6). Immediately preoperative weight at revision, the mean weight decreased to 87.7 kg (median 87.0, range 48–158), and BMI averaged 32.2 (median 33.0, range 18.5–51.9). Following revision, the mean weight further declined to 75.2 kg (median 74.0, range 41.0–129.0), with a BMI of 27.7 (median 27.5, range 15.6–40.7). Overall, weight decreased by an average of 44.6 kg from pre-BPD to pre-revision and by an additional 13.0 kg after revision, while BMI decreased by 16.4 points before revision and 4.7 points after revision (Fig. 1). The follow-up (FUP) duration had a mean of 7.3 months, a median of 6.0 months, and ranged from 1 to 12 months.

### Type and Indications for Revision

The most frequent indication was Malnutrition/Malabsorption (22 cases, 47.8%), followed by Weight regain (17 cases, 37.0%) and combined reasons (6 cases, 13.0%).

As for type of revision, the leading one was common tract lengthening through biliopancreatic limb shortening (13 cases, 28.3%), followed by gastric pouch trimming(14 cases, 30.4%). Combined procedures involving both limbs resizing and pouch reshaping accounted for 14 cases (30.4%). Conversions to RYGB were performed in 2 cases (4.3%), and conversion to OAGB in 1 case (2.2%). Other complex revisions represented 2 cases (4.3%).

Five (10.9%) patients underwent a second revision. The most common procedure was gastric pouch resizing (3 cases, 60.0%), followed by bipartition with gastroileal anastomosis (1 case, 20.0%) and common channel adjustment (1 case, 20.0%).

The median length of stay was 4.0 days, with a range of 2–77 days.

### Surgical Approach

Among the index BPD procedures, 26 cases (56.5%) were managed by a laparoscopic approach (LPS), whereas 20 cases (43.5%) underwent open surgery (LPT). Regarding revisions, 33 cases (71.7%) were performed laparoscopically, while 13 cases (28.3%) were open. Of the 20 patients with initial open surgery, 6 (30%) underwent a laparoscopic revision. Conversely, of the 26 patients with initial laparoscopic BPD, 3 (11.5%) required an open revision. Most second revisions were performed laparoscopically (3 cases, 60.0%), while 2 cases (40.0%) required open surgery.

All patients underwent multidisciplinary evaluation and those suffering from malnutrition required protein restoration and micronutrient repletion, with protein intake targeted at ≥ 1.0–1.5 g/kg/day while deficiencies were corrected through IV iron, high-dose fat-soluble vitamins, and calcium citrate with vitamin D. Patients with hypoalbuminemia often needed oral or enteral supplementation to stabilize levels before surgery. As a result, surgery *was* delayed when necessary to ensure patients entered the operating room in a safer, nutritionally replete state.

## Functional Outcomes

**Fig. 2:**
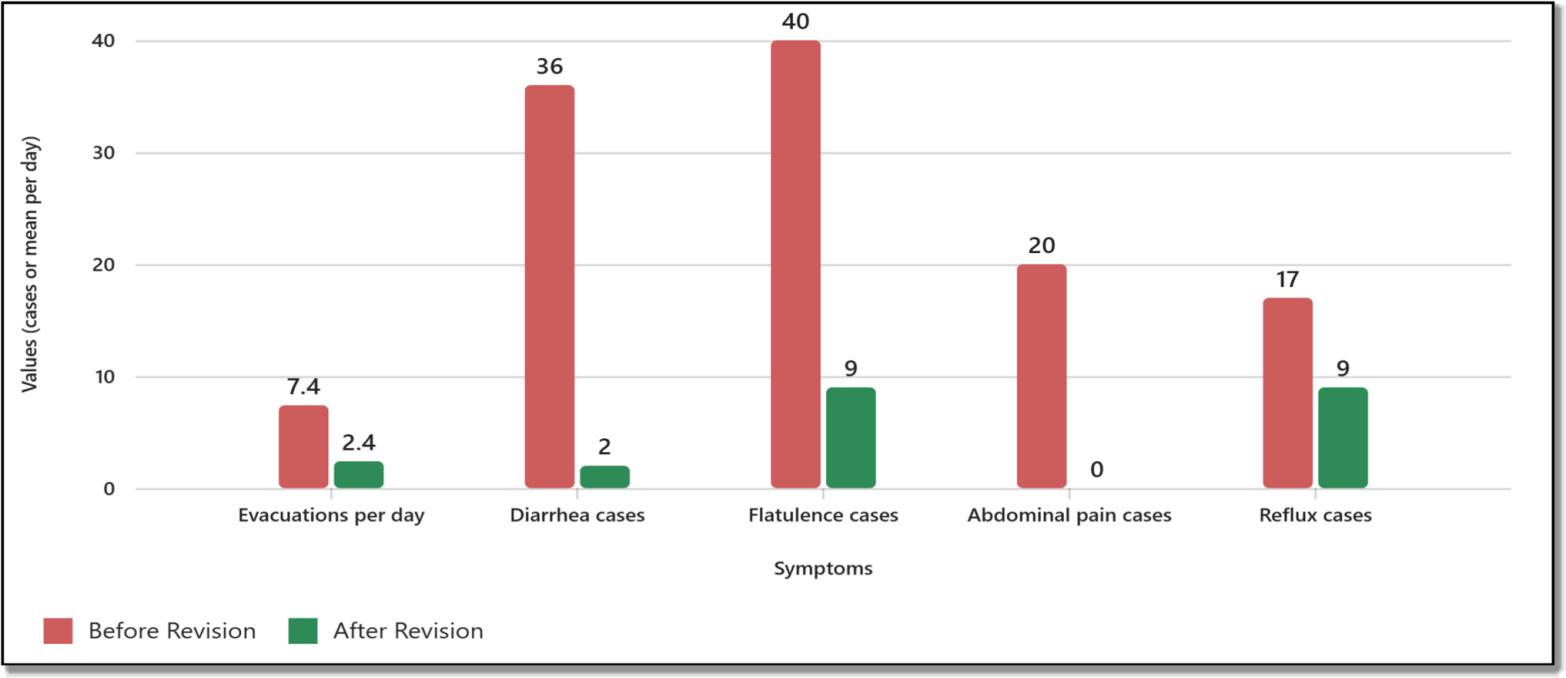
Comparison of symptom prevalence and mean daily evacuations before and after surgical revision

The mean number of evacuations per day decreased markedly from 7.4 (range 1–30) pre-revision to 2.4 (range 1–5) post-revision (Wilcoxon signed-rank test, p < 0.0001). Symptom prevalence also declined substantially (McNemar tests): diarrhea dropped from 80.0% to 4.4% (p < 0.0001), flatulence from 90.9% to 20.5% (p < 0.0001), and abdominal pain resolved completely (45.5% to 0%, p < 0.0001). Reflux improved from 38.6% to 20.5% (p = 0.013). Gastrointestinal symptoms pre- and post-revision are also pictured in Fig. 2.

### Complications

Post-revision complications occurred in 9 out of 46 patients (19.6%, Table 1).

**Table 1:** Early and late Complications

Category	Complication	Cases (n = 46)/Percentage
Early Complications	Total early complications	9 (19.6%)
	Managed via laparotomy	8 (17.4%)
	Managed via laparoscopy	1 (2.2%)
	Mortality	1 (2.2%)
Complications at Last Follow-up	Anemia requiring treatment	26 (56.5%)
	Protein malnutrition	15 (32.6%)
	Kidney stones	15 (32.6%)
	Ulcers	1 (2.2%)

Intestinal occlusion occurred in 3 cases (6.5%), followed by volvulus in 1 case (2.2%) and anastomotic dehiscence in 1 case (2.2%). Other severe complications included splenic necrosis with pancreatic fistula (1 case, 2.2%), occlusion associated with perianastomotic hematoma (1 case, 2.2%), jejuno-jejunal leak requiring open abdomen (1 case, 2.2%), and evisceration with entero-enteral anastomotic dehiscence (1 case, 2.2%). Most complications were managed via laparotomy (8 cases, 17.4%), while laparoscopy was used in 1 case (2.2%). Mortality occurred in the patient with a jejuno-jejunal leak (2.2%).

The most common complication at Last Follow-upwas treatment-requiring anemia (26 cases, 56.5%), followed by protein malnutrition (15 cases, 32.6%) and kidney stones (15 cases, 32.6%). Ulcers were rare, occurring in 1 case (2.2%).

## Discussion

Revisional surgery represents just over 10% of all bariatric procedures worldwide, with gastric bypass being the most frequently performed revisional operation worldwide and in Italy [7, 8]. Yet, because BPD was so successful and widely adopted in the past, high-volume centers today must be equipped to handle patients who, although infrequently, present with indications for surgery.

Indeed, numerous long-term studies consistently show that a non-negligible revisional rate is an inherent aspect of the BPD procedure.

A spanish study [9] evaluated 277 Scopinaro BPD patients over a 10-year follow-up, comparing open (n = 205) and laparoscopic (n = 72) approaches. Although weight-loss outcomes were similar, laparoscopy significantly reduced hospital stay (4.6 vs. 9.7 days, p = 0.04) and markedly lowered the incidence of incisional hernias (5.5% vs. 27.8%, p = 0.04). Long-term protein malnutrition occurred in 4.5% of patients, with 2.8% requiring surgical elongation of the intestinal limbs.

A Brazilian center[10] followed 1,570 patients for up to 20 years after Scopinaro’s biliopancreatic diversion with gastric preservation. Overall, 13% developed late complications, and 9.1% required reoperation, while 29.9% were managed clinically with good outcomes. The most frequent issues were malnutrition (46.5%), chronic diarrhea (28.4%), gastroileal ulcers (21.5%), and severe anemia (20.5%). Surgical management included common channel elongation (4%), conversion to gastric bypass (5%), and restoration of normal anatomy (0.6%), with only one death (0.06%) reported.

A review by Topart[11] reported that Scopinaro BPD was associated with a high revision rate, ranging from 3% to 18.5%, while reversal was required in 2.1–7% of cases. Reversal was predominantly indicated for protein malnutrition, which accounts for approximately 43–60% of all reoperations.

Later, Ceriani et al. [12] suggested a combined revision—extending the common channel to 200 cm and reducing the gastric pouch to 40 ml—which proved effective for both proctologic symptoms and inadequate weight loss after Scopinaro BPD. In 38 patients, bowel movements dropped from 3 to 1 per day and weight decreased from 87.1 kg to 69.2 kg at 1 year, remaining stable at 5 years. Nutritional status stayed largely preserved, with no recurrence of protein malnutrition.

Our series highlighted the long-term trajectory of patients who required revisional surgery after biliopancreatic diversion. Revisions occurred at a mean of nearly 18 years, at a time when patients had already achieved a substantial reduction from their pre-BPD weight—underscoring that although BPD produced durable weight-loss effects, late complications still emerged in a subset of individuals. Notably, revisional surgery was associated with an additional and clinically meaningful reduction in BMI, reinforcing its therapeutic value when late failure or complications occurred.

The pattern of our revisions reflected the typical long-term vulnerabilities of biliopancreatic diversion, with nearly half of reinterventions performed for malnutrition or malabsorption and over one-third for weight regain. Surgical strategies varied widely, ranging from common channel lengthening to gastric pouch reshaping, with many patients requiring combined limb and pouch modifications. Conversions to RYGB or OAGB were rare but reserved for complex cases.

Our findings highlighted a gradual evolution in surgical practice over time, showing that both laparoscopic and open approaches continued to be used depending on patient characteristics and procedural complexity. Despite this shift, revisional surgery after BPD remained technically demanding, as nearly one-third of revisions still required an open approach, often due to dense adhesions or altered anatomy. Notably, a second revision was needed in about 11% of patients, and these re-reoperations frequently demanded open access, underscoring the limits of minimally invasive techniques in heavily reoperated fields.

Complications occurred in almost 20% of patients, with severe events such as intestinal occlusion, volvulus, anastomotic dehiscence, and even splenic necrosis, most of which required laparotomy for definitive management. The single mortality following a jejuno-jejunal leak underscored the high-risk nature of these reoperations. The complication profile shows that anemia was the most frequent late issue, affecting over half of the cohort and reflecting the profound malabsorptive burden of classic BPD. Protein malnutrition and remained in 32.6% of patients despite revision, underscoring that even corrective surgery cannot fully eliminate the long‑term nutritional and metabolic vulnerabilities inherent to this procedure.

Overall, these findings illustrated that revisional surgery after BPD was not only technically complex but also associated with meaningful perioperative and long-term challenges, reinforcing the need for specialized expertise and lifelong follow-up in this patient population.

However, Revisional surgery produced a clear functional benefit, with bowel frequency dropping from more than seven to just over two evacuations per day. Debilitating symptoms such as diarrhea, flatulence, and abdominal pain improved dramatically, with abdominal pain resolving completely and diarrhea nearly disappearing. Even reflux, typically less responsive to anatomical revision, showed significant improvement. Overall, these findings showed that revision after BPD not only corrected structural or metabolic issues but also restored far more acceptable gastrointestinal function, markedly improving patients’ daily quality of life.

### Limitations

The retrospective design, the relatively small sample size, and the limited postoperative follow‑up restricted the ability to draw long‑term conclusions. However, contemporary literature on revisional surgery after classic BPD is scarce, which makes these findings particularly valuable in helping to fill an important gap in current evidence. Beyond anatomical and metabolic contributors, patient-related factors may also influence outcomes after BPD. Limited adherence to long-term follow-up and the presence of untreated psychiatric comorbidities can contribute to late failure or the eventual need for revisional surgery[13]. Although these aspects were not systematically assessed in the present cohort, they represent important determinants of nutritional management, compliance with supplementation, and long-term weight trajectories, and should be incorporated into future analyses to better characterize the full spectrum of risk factors associated with post-BPD deterioration[14].

## Conclusions

Both malnutrition and weight regain emerged as the leading indications for revision after BPD, reflecting the long-term metabolic vulnerability of this procedure. Revisional surgery proved technically demanding but was increasingly feasible through a laparoscopic approach. Nonetheless, the severity of postoperative complications—often requiring laparotomy, re-intervention, or even resulting in mortality—indicated that these operations should remain confined to experienced, high-volume centers. The marked improvement in gastrointestinal function after revision confirmed its therapeutic value when appropriately indicated. Overall, these findings underscored the importance of careful patient selection and lifelong follow-up for individuals who previously underwent BPD.

## Data Availability

No datasets were generated or analysed during the current study.
